# Removal of Pharmaceuticals from Water by Free and Imobilised Microalgae

**DOI:** 10.3390/molecules25163639

**Published:** 2020-08-10

**Authors:** Telma Encarnação, Cátia Palito, Alberto A. C. C. Pais, Artur J. M. Valente, Hugh D. Burrows

**Affiliations:** Centro de Química de Coimbra CQC, Department of Chemistry, University of Coimbra, 3004-535 Coimbra, Portugal; catia_palito@hotmail.com (C.P.); pais@qui.uc.pt (A.A.C.C.P.); avalente@qui.uc.pt (A.J.M.V.); burrows@ci.uc.pt (H.D.B.)

**Keywords:** immobilisation, pharmaceuticals, endocrine disrupting chemicals, bioremediation, pollutants removal, polyvinyl alcohol

## Abstract

Pharmaceuticals and their metabolites are released into the environment by domestic, hospital, and pharmaceutical industry wastewaters. Conventional wastewater treatment technology does not guarantee effluents of high quality, and apparently clean water may be loaded with pollutants. In this study, we assess the performance and efficiency of free and immobilised cells of microalgae *Nannochloropsis* sp. in removing four pharmaceuticals, chosen for their occurrence or persistence in the environment. These are paracetamol, ibuprofen, olanzapine and simvastatin. The results showed that free microalgae cells remain alive for a longer time than the immobilised ones, suggesting the inhibition of cell proliferation by the polymeric matrix polyvinyl alcohol. Both cells, free and immobilised, respond differently to each pharmaceutical. The removal of paracetamol and ibuprofen by *Nannochloropsis* sp., after 24 h of culture, was significantly higher in immobilised cells. Free cells removed a significantly higher concentration of olanzapine than immobilised ones, suggesting a higher affinity to this molecule than to paracetamol and ibuprofen. The results demonstrate the effectiveness of *Nannochloropsis* sp. free cells for removing olanzapine and *Nannochloropsis* sp. immobilised cells for removing paracetamol and ibuprofen.

## 1. Introduction

Besides air pollution, water pollution is one of the most important environmental problems that the world urgently needs to address, since clean water is a vital resource for every living organism on the planet. The water of oceans, seas, rivers and lakes have natural mechanisms of self-cleaning that rely on plankton, which is also the base of food-chains and food-webs. They remove the waste and contaminants produced by other organisms in aquatic ecosystems, such as carbon dioxide, nitrates and phosphates. They also remove the non-natural anthropogenic contaminants, produced by humans, such as pesticides, industrial chemicals and pharmaceuticals. However, there are certain threshold levels, above which these mechanisms start to fail. Anthropogenic contaminants reach water bodies through different pathways: direct sewage effluent discharges, via rivers, urban and agricultural run-offs, dumpings, atmospheric transport, and various other means. According to the United Nations Environment Programme (UNEP), 80% of global wastewater is released untreated into water bodies [1]. Pharmaceuticals and their metabolites are released to the environment by domestic and hospital wastewaters and by the pharmaceutical industry wastewater [2]. Pharmaceuticals and their metabolites are also excreted through urine and faeces into sewage. The metabolic path of some pharmaceuticals, containing up to 95% of the active ingredient, may involve excretion in the original form [3]. These are eventually discharged into water bodies through municipal wastewater.

In general, these are reported at the range of nanogram to microgram per litre, while some have been detected up to the range of milligrams per litre [4]. Pharmaceuticals are designed to be biologically active, and to trigger a specific therapeutic response in humans or animals, based on biology and size. The concentrations that are innocuous to humans could be deadly to non-target organisms, which share certain homologous receptors with humans. Certain pharmaceuticals, such as ethinyl oestradiol, diclofenac and paracetamol, are known to cause endocrine disruption in the organisms [5,6]. Increasing evidence is emerging on the implications of endocrine disrupting chemicals in the environment and wildlife, and on human health, at very low doses [7]. Effects on biota have been reported at concentrations in the range of ng per litre [8,9]. The dimension of the problem requires global action. 

On March 2019, the European Commission released the European Union Strategic Approach to Pharmaceuticals in the Environment, contributing to achieving the Sustainable Development Goal 6, on clean water and sanitation [10]. This Communication defines a strategic approach to addressing the global problem of pharmaceuticals in the environment. It calls for more advanced wastewater treatment technologies and more research, and encourages innovation “where it can help to address the risks, and promote the circular economy by facilitating the recycling of resources such as water, sewage sludge and manure” maintaining “the access to safe and effective pharmaceutical treatments for human patients and animals”. 

Given the low concentration of drugs in wastewaters and their nature, such as hydrophilicity, solubility, volatity and biodegradability [4], the conventional wastewater treatment plants are not, in general, effective for their removal. For example, Gao et al. have reported the removal rate of pharmaceuticals in different sewage treatment plants [11]. They have found that paracetamol had a removal rate of >99% at the end of the entire wastewater treatment processes. However, the highly persistent antibiotic trimethoprim had relatively low removal efficiency, in the range of 9%–40%. Therefore, several approaches have been developed to overcome this shortage. They include the use of Advanced Oxidation Processes (AOPs), including, e.g., ozonation and photo-Fenton reactions, have been used for the treatment of pharmaceuticals-containing wastewaters [4]. Another way to remove drugs from wastewater is by adsorption. A multitude of adsorbents have been successfully tested. They include activated carbon [12,13], magnetic nanoparticles [14], aerogels [15,16], or hybrid systems [17]. 

Despite this, the application of green technologies such as Constructed Wetlands (CWs) could be applied effectively with high performance for the removal of conventional and emerging pollutants [18]. CWs are engineered land-based wastewater treatment systems designed to mimic the functions of natural wetlands. This technology can be applied to several types of industrial wastewater, such as petrochemical, chemical, paper and pulp, textile and dairy industry wastewater sources [19]. The reported removal efficiency of some pharmaceuticals in CWs are in the range of nanogram to microgram per litre [19]. CWs present many advantages such as low operational cost, simple operation and maintenance and high performance. However, this technology presents some drawbacks, including progressive clogging, biofilm growth, plant decay products, chemical precipitation, and large areas of land being required [19]. 

Another potential green technology, which could be combined with the currently used wastewater treatment plants, and does not present the disadvantages of CWs, is microalgae technology. It is well known that microalgae remove nitrates and phosphates from wastewaters. They need these nutrients for their growth. Bioremediation using microalgae presents several advantages, including high biomass productivity, high carbon uptake and bioconversion, not requiring large areas of land, and a biorefinery concept that can be used for the production of chemicals from the microalgal biomass. There are already companies that are successfully using microalgae technology to remove heavy metals or to recover nutrients from wastewater, adding value to the resulting biomass [20,21]. High capital investment and operating costs are the most important disadvantages associated with bioremediation technology.

The majority of the bioremediation studies focus on the removal of nitrates, phosphates and heavy metals from wastewaters and sewage effluents. However, studies on emerging pollutants, such as pharmaceuticals, are very scarce. 

In line with the European Commission’s strategy, and with Sustainable Development Goal 6, in this study we assess the performance and efficiency of free and immobilised cells of microalgae *Nannochloropsis* sp. for the removal of four pharmaceuticals, chosen for their occurrence or persistence in the environment. These are paracetamol (PAR), ibuprofen (IBU), olanzapine (OLA) and simvastatin (SIM). 

Acetaminophen, commonly known as paracetamol (PAR), is a widely used analgesic and antipyretic drug, and is known to be heavily present in the aquatic environment. Ibuprofen is a non-steroidal anti-inflammatory drug, commonly used for the relief of pain, fever and inflammation. Both are generally classified as harmful to aquatic organisms [22]. Olanzapine, an antipsychotic drug, used for the treatment of schizophrenia, is resistant to photodegradation by sunlight, and is found in surface waters [23,24]. Simvastatin is a lipid-lowering drug, known for anti-hyperlipidemic activity. This substance is one of the most commonly used prescriptions worldwide, and is, therefore, of environmental concern. This pharmaceutical compound is a lactone that is hydrolysed in water to the corresponding β-hydroxyacid, denoted simvastatin acid (SIMA). 

In bioremediation research, the microalgae genus *Chlorella* are the most commonly used ones. The species selected for this study was *Nannochloropsis* sp. This species was chosen for the uptake experiments because it possesses a high growth rate, resilience, adaptation to a wide range of growth media, halotolerance, and accumulation of large amounts of lipids, which is an advantage as it ads value to the biomass produced during the bioremediation process (biorefinery concept). Therefore, the aim of this study is to assess the removal efficiency of four pharmaceuticals by the microalgae *Nannochloropsis* sp. Two different approaches were compared: the removal of pharmaceuticals by free cells, and the removal of pharmaceuticals by immobilised cells. The polymeric matrix selected for the immobilisation experiments was poly(vinyl alcohol) (PVA). This gel matrix was selected because it is considered to be biocompatible and non-toxic, and displays good mechanical properties [25].

## 2. Materials and Methods

### 2.1. Reagents and Chemicals

Paracetamol (PAR) was purchased from Fagron Iberica (Zaragoza, Spain) and Ibuprofen (IBU) was supplied by Laboratórios Medinfar (Lisboa, Portugal). Olanzapine (OLA) was acquired from Zhejiang MYOY Import and Export Co., Ltd. (Hangzhou, China). Simvastatin (SIM) was kindly provided by Labesfal, Laboratórios Almiro, S.A. (Santiago de Besteiros, Portugal). Microalgae medium f/2 was obtained from Varicon Aqua Solution (Malvern, UK). Poly(vinyl alcohol) (PVA), having a molecular weight of 61.000, commercial name Mowiol^®^ 10–98, was obtained from Aldrich Chemistry. Boric acid (H_3_BO_3_), with an average molecular weight of 61.83, was purchased from Sigma-Aldrich (India). Calcium chloride (CaCl_2_), with a molecular weight of 110.99, was acquired from Merck. All other reagents and solvents were of analytical or HPLC grade.

### 2.2. Organism and Culture Conditions

*Nannochloropsis* sp. was obtained from Varicon Aqua Solution, Malvern, UK, and was cultivated for 6 days in 2 L f/2 medium. 100 cm^3^ of a *Nannochloropsis* sp. culture were filtered and washed. The cells with a concentration of about 2 × 10^7^ cell cm^−3^ were subsequently transferred to a closed photobioreactor, to which 100 cm^3^ of culture medium Cell-hi TEViT (Varicon Aqua Solution, Malvern, UK) was added, based upon the f/2 medium, deprived of nitrates. The nitrate concentration was 0.30 g L^−1^ and the salinity was 25 g L^−1^. The culture was aerated by bubbling atmospheric air, at a rate of 300 cm^3^ min^−1^, were grown at 25 ± 2 °C under light with an irradiance level of ± 100 μmol m^−2^s^−1^ with 16:8 photoperiod and kept for 60 h. Non-sterilized Milipore water was added when needed to ensure the same constant volume, compensating for water loss by evaporation. Samples of 5 mL were replaced with ultrapure water. Each experiment was carried out in triplicate. The cellular density was determined using the Neubauer chamber (hematocytometer) in an optical microscope. The supernatant (filtrate culture medium) was collected, filtered, and stored at −20 °C until analysis.

### 2.3. Fluorescence Microscopy Observation

Fluorescence images of *Nannochloropsis* sp. cells were obtained using an Olympus BX51 M microscope, equipped with a UplanFL N 100x/1.30 oil immersed objective lens (∞/0.17/FN/26.5), a filter set type U-MBF3, U-MWV2, and an UV-mercury lamp (100W Ushio Olympus). Images were digitized on a computer through a video camera (Olympus digital camera DP70) and were analysed with an image processor (Olympus DP Controller 2.1.1.176, Olympus DP Manager 2.1.1.158). The observations were carried out at room temperature (≈25 °C). 

### 2.4. Immobilization Procedures

**Figure 1:**
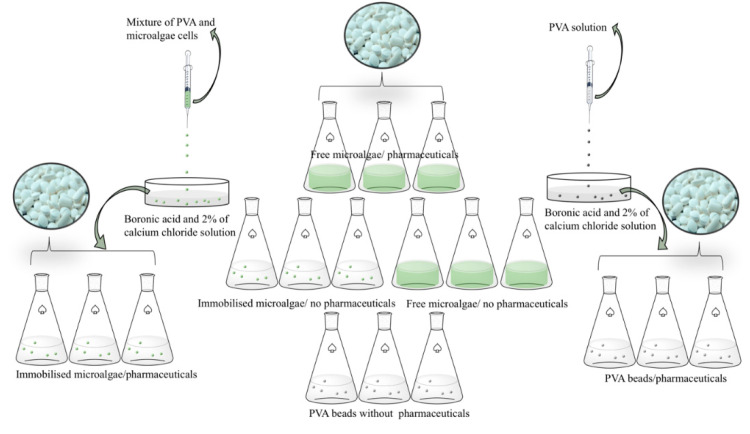
Schematic representation of the procedure used in the bioremediation experiments.

The immobilization of the microalgae was achieved through the formation of beads of PVA gel. A 24% PVA solution was first prepared and dissolved with continuous stirring at about 50 °C for two hours. In parallel, the microalgae were centrifuged and washed to remove media salt and nutrients, and then added to the PVA solution in a 1:2 portion (cells: PVA). This mix was dripped with a sterile syringe into a saturated solution of boronic acid and 2% of calcium chloride in which the beads formed remained for one hour. As a control, beads were prepared consisting of only PVA. In parallel, the solutions with the pharmaceuticals were prepared (Figure 1).

In the preparation of the beads, 2 mL of PVA and 1 mL of microalgae cells yielded approximately 25 beads, each ca. 4 mm in diameter. The structure of the cell wall of the microalgae was composed of various compounds such as polysaccharides, proteins, and lipids. These compounds contained functional groups, including amino, carboxyl, sulphate, phosphoryl and hydroxyl groups. As a result, the microalgae membrane was negatively charged. The functional groups present in the polymeric matrices interact with the functional groups of the microalgae cell wall, forming both covalent and hydrogen bonding, and also Van der Waals interactions [26].

### 2.5. Instrumentation and Chromatographic Conditions

The chromatographic analysis of PAR, IBU, OL, SV and SVA was carried out using a Dionex Ultimate 3000 system equipped with an auto injector and four variable UV/visible dual wavelength detectors. The column used for the analysis was a Luna Phenyl-Hexyl, Phenomenex^®^ (Torrance, CA, USA), with 5 μm particle size, 3 mm internal diameter and 150 mm length, supported with a SecurityGuard™ cartridge Phenomenex^®^ (Torrance, CA, USA), with a 3.0 mm internal diameter, in an oven at a temperature of 35 °C. The results were acquired and processed using Chromeleon software. The mobile phase consisted of a mixture acetronitrile (eluente A) and 0.01M dipotassium hydrogen phosphate aqueous solution (eluent B), at a pH 7.3 ± 0.1 and constant flow rate of 0.8 mL/min. Chromatographic analysis was conducted with a multistep gradient mode (0 min 70% Eluent B, 1 min 60% Eluent B, 2 min 40% Eluent B, 5 min 35% Eluent B, 7 min 30% Eluent B, 8 min 70% Eluent B). 

### 2.6. Method Validation

The method for PAR, IBU, OLA, SIM, and SVA quantification was validated according to the US Food and Drug Administration (FDA), the International Conference on Harmonization (ICH) guidelines, and the Eurachem, with respect to system suitability, linearity, accuracy, precision, recovery, limits of detection and quantification, selectivity and specificity. The optimization and validation details and results are presented in the study by Encarnação et al. [27]

### 2.7. Experimental Design

Different simultaneous sets of experiments were conducted at 25 ± 2 °C, under light with an irradiance level of ± 100 μmol (Figure 1). A concentration of 50 μg mL^−1^ of each pharmaceutical, PAR, IBU, OLA, SIM and simvastatin acid (SIMA), was added to the 100 cm^3^ free cells *Nannochloropsis* sp. cultures. Similarly, a blank sample with 50 μg mL^−1^ of each pharmaceutical, PAR, IBU, OLA, SIM and SIMA, was added to 100 cm^3^ of culture medium f/2, without cells. The experiments with cells in beads were performed in the same way; the immobilised cells *Nannochloropsis* sp. cultures, the beads, were transferred to the 100 cm^3^ of culture medium f/2. The control experiment was carried out with beads in 100 cm^3^ of culture medium f/2, but in the absence of cells. All the experiments had a 16:8 photoperiod and were run for 60 h. Millipore water was added when needed in order to ensure constant volume in the presence of water loss by evaporation. Samples of 5 mL were replaced with Millipore water. The cultures were aerated by bubbling atmospheric air, at a rate of 300 cm^3^ min^−1^, and kept for 60 h. The samples were collected after 12, 24, 36 and 60 h, filtered and analyzed by RP-HPLC. Each experiment was carried out in triplicate.

### 2.8. Mathematical Correction

Since the determination of the concentration of each pharmaceutical relies on the sampling of a dynamic system, with the maintenance of the working volume and changes in the population, an erroneous interpretation of the system is frequent. Therefore, a mathematical correction is necessary and is one that compensates for the dilution effects. The following equation was applied

(1) $${P^{\prime }}_{t2}=P_{t1}-\left(P_{t1}\times \frac{95}{100}-P_{t2}\right)$$

where ${P^{\prime }}_{t2}$ is the corrected concentration (μg mL^−1^) of the drug on time 2, $P_{t2}$ is the enhanced concentration (μg mL^−1^) of the pharmaceutical on time 2 and $P_{t1}$ is the enhanced concentration (μg mL^−1^) of the pharmaceutical on time 1. This equation was applied to correct each reading after the method validation. 

### 2.9. Statistics

A single factor ANOVA analysis was conducted to establish the statistical significance within each treatment. A probability level of <0.05 was used as the criterion for null hypotheses. This analysis was performed using Microsoft Excel^®^ (Microsoft Corp., Redmond, WA, USA).

## 3. Results and Discussion

### 3.1. Evaluation of Resistance toward Pharmaceuticals in Nannochloropis Sp.

**Figure 2:**
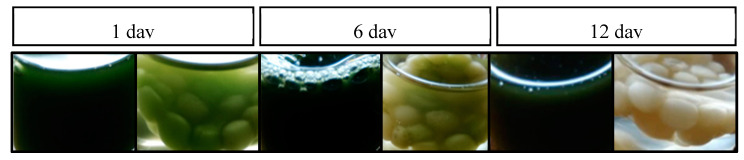
Free (left) and immobilised *Nannochloropsis* sp. cells (right) grown in f/2 medium and in the presence of PAR, IBU, SIM and OLA, for the indicated periods.

**Figure 3:**
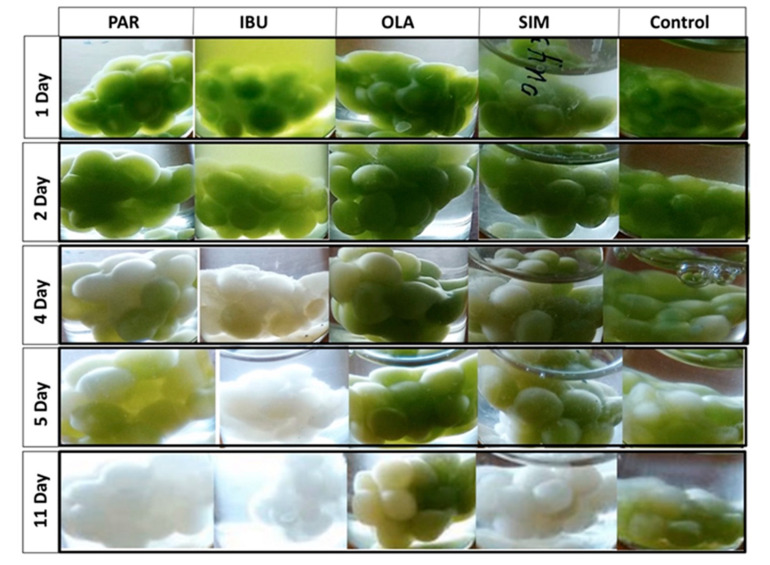
Immobilised *Nannochloropsis* sp. cells grown in f/2 medium and PAR, IBU, SIM and OLA, separately, during 11 days of culture.

The *Nannochloropsis* sp. cells were efficiently immobilized in the PVA matrix. Preliminary studies were carried out with free and immobilised cells in an f/2 medium solution where 100 μg mL^−1^ of each pharmaceutical had been added (Figure 2), and with each pharmaceutical separately (Figure 3). From Figure 2, it can be seen that the free cells, in the presence of the four pharmaceuticals, remained a viable population for at least 12 days. In contrast, immobilised cells started to fade from the sixth day onwards, and no viable cells were observed at the end of the experiment. 

The preliminary results showed that the immobilisation matrix of the PVA affected the microlagae population and promoted a negative effect on the survival of microalgal cells. It also indicated that *Nannochloropis* sp. was a resilient species to exposure to studied drugs.

In the evaluation of each individual pharmaceutical, Figure 3 shows that the most harmful pharmaceutical to the immobilised cells was ibuprofen. After four days, cells were completely faded, indicating the breakdown of the culture. The control group (no pharmaceuticals added to the medium) showed some fading after four days. The immobilised cells grown in the presence of olanzapine showed a higher resistance.

Comparing the exposure of immobilised cells to different pharmaceutical drugs, separately and together, it can be concluded that the cells showed longer viability in the presence of the four drugs. Previous reports have demonstrated that microalgae remove different pollutants with different efficiencies [28,29]. The extent of removal depends on the microalgae strain used, the nature of the pollutant and its concentration in the medium. Furthermore, interferences of interactions between compounds and the effect of the matrix on cell metabolism can be a plausible explanation for the different cell responses observed in Figure 2 and Figure 3. 

### 3.2. Fluorescence Microscopy Observation

**Figure 4:**
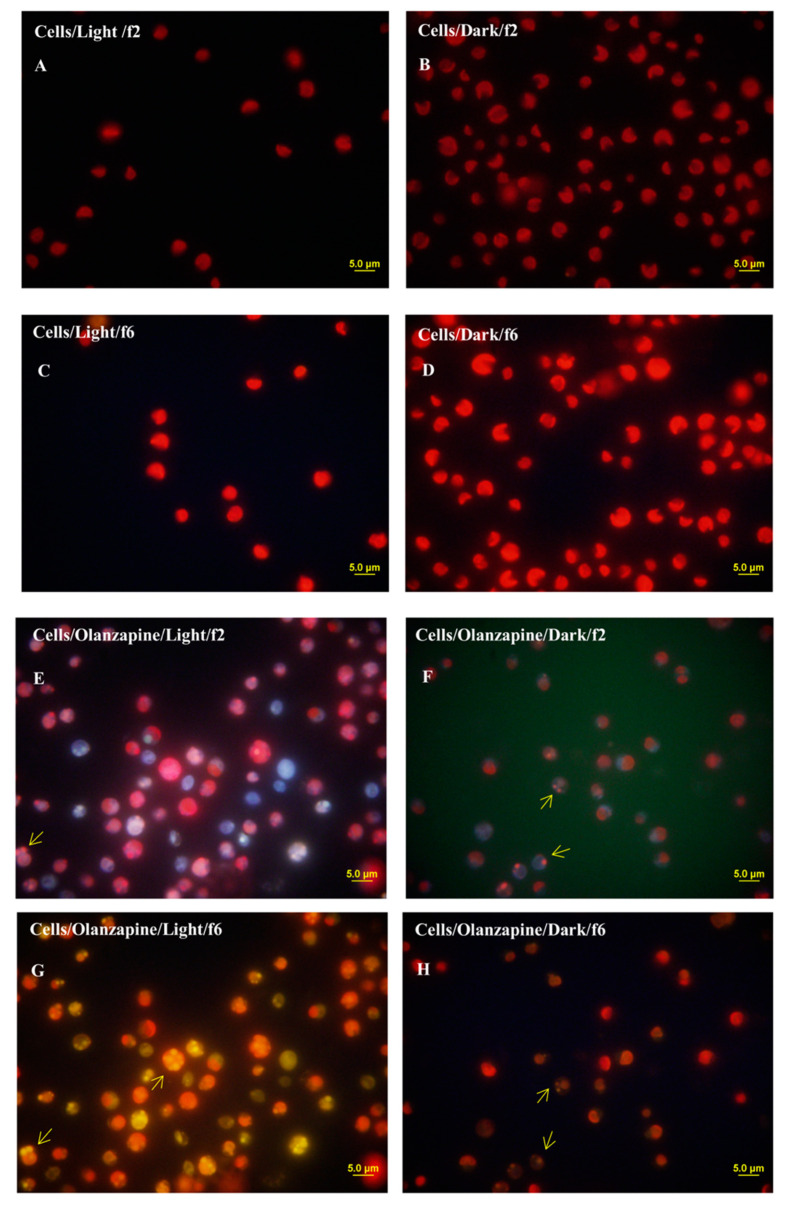
Epifluorescence microscopy images of *Nannochloropsis* sp. cells showing the red autofluorescence of chlorophyll from chloroplasts and fluorescence of the olanzapine. Cells grown in an f/2 medium (no pharmaceuticals added to the medium) (**A**–**D**). (**A**,**C**) show cells grown autotrophically. (**B**,**D**) show cells grown in the dark (different microscope filters, f2 and f6). Cells grown in f/2 medium with OLA added to the medium (**E**–**H**). (**E**,**G**) cells grown autotrophically (microscopy filters f2 and f6). (**F**,**H**) cells grown in the dark (microscopy filters f2 and f6).

The marine species *Nannochloropsis* sp. possesses a characteristic cell structure; the cell relies on a big chloroplast that occupies more than half of the cell when grown in nitrogen replete medium. The chloroplasts of the cells emitting the characteristic red fluorescence of chlorophyll were observed in Figure 4. In an f/2 medium, without the presence of pollutants, the characteristic emission of fluorescence of cells resembles what can be observed in Figure 4A–D. However, in the presence of olanzapine, fluorescence was observed as following an internalization process in the living cells, Figure 4E–H. Differences can be discerned between cells grown autotrophically and cells grown in the dark. The marine species *Nannochloropsis* sp. was capable of both heterotrophic growth in the dark and phototrophic growth in the light. During phototrophic growth, microalgae cells harvested light energy, captured CO_2_ as a carbon source, and produced O_2_. Microalgae cells grown in the dark consumed O_2_ and produced CO_2_. As expected, the metabolic pathways changed. The metabolism of microalgae cells changed depending on many factors, such as oxygen availability and the nature of the carbon source, in the case of heterotrophic growth, and on the CO_2_ availability and amount of light energy received, in the case of phototrophic growth. Therefore, these changes in cellular metabolism modulated the removal of pollutants from the growth media.

### 3.3. Population Density

**Figure 5:**
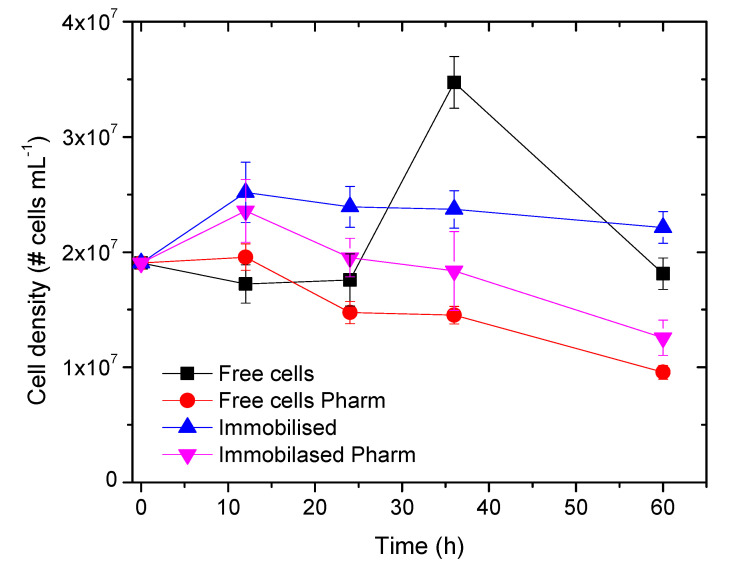
Growth curve of *Nannochloropsis* sp. in continuous cultures for 60 h. Error bars represent SD. The lines are guides for the eyes.

Cultures of *Nannochloropsis* sp. immobilized in PVA beads grew from 1.9 × 10^7^ cells mL^−1^ to 2.4 × 10^7^ cells mL^−1^ in the first 12 h and remained stable, with a slight decrease after 60 h of culture, 2.2 × 10^7^ cells mL^−1^ (Figure 5). Cultures of *Nannochloropsis* sp. immobilized in PVA and grown in the presence of the pharmaceuticals had a similar growth in the first 12 h and then decreased continuously until the end of the experiment.

The highest growth was obtained in free cells without the presence of drugs, during which, after 36 h of culture, the population reached 3.5 × 10^7^ cells mL^−1^. The lowest growth, a cell density of 9.6 × 10^7^ cells mL^−1^ after 60 h, was observed with the free cells grown in the presence of pharmaceuticals. While PVA was considered to be biocompatible, the experiments showed that it inhibited cell proliferation (see immobilised cells without pharmaceuticals). The lower growth rates could have been due to limitations in the diffusion of nutrients through the bead structure. However, it is interesting to note that the population remained stable over time, in contrast to the free cells without pharmaceuticals. In this culture the free cells had all the nutrients available for consumption. After depletion of all the nutrients, the population decreased.

### 3.4. Removal of Pharmaceuticals by Nannochloropsis *Sp.*

Upon analysis by Rapid Reversed-Phase High Performance Liquid Chromatography (RP-HPLC), the chromatograms revealed the presence of PAR, IBU and OLA; SIM was not detected. To check the possibility of precipitation of the compound, a filtrate was extracted with acetonitrile and analysed by RP-HPLC. The chromatogram revealed the presence of SIM in its original concentration, confirming its precipitation. For this reason, this study only focused on PAR, IBU and OLA. 

**Figure 6:**
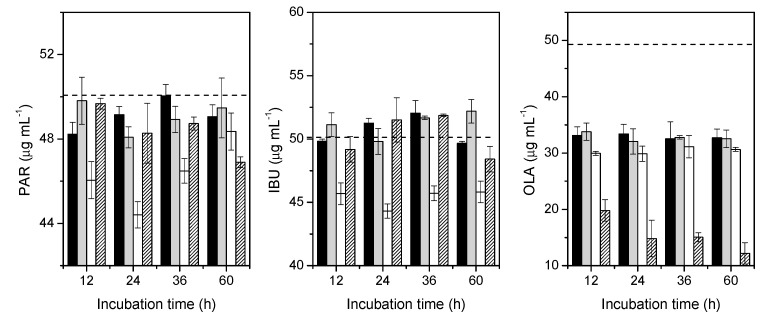
PAR, IBU and OLA removal by *Nannochloropsis* sp. in continuous cultures for 60 h. f/2 medium (black), Beads (gray), Immobilized (white) and Free cells (black lines). Horizontal dashed lines represent the concentration at t = 0. The chromatogram shows an overestimated determination for IBU concentrations that corresponds to expected fluctuations in the measurements (the error in the concentration determination is around 4%). Bars represent the SD.

**Figure 7:**
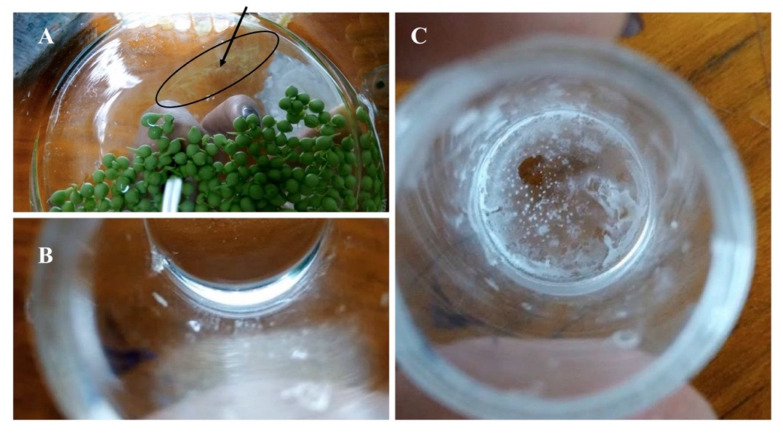
Evaluation of the integrity of the Poly(vinyl alcohol) (PVA) beads. (**A**) Leakage of cells from the beads, after 7 days of being prepared. (**B**) Filtrated solution (f/2 medium and addition of pharmaceuticals) of the control group, after the removal of the beads of PVA without cells. (**C**) Evaporation of the water, a film of PVA settled at the bottom of the glass beaker.

Figure 6 shows that the removal of PAR by *Nannochloropsis* sp. after 24 h of culture was significantly higher in immobilised cells, from 50.5 to 44.4 μg mL^−1^, with a *p* value = 0.05. In the f/2 medium, beads and free cells did not influence the concentration of PAR. However, the concentration of PAR in the group with the immobilised cells apparently showed an increase from 44.4 to 48.4 μg mL^−1^ after 60 h of culture. However, upon closer examination (Figure 7), it appears that some fraction of the PVA beads had dissolved, with the probable release of the PAR into the medium. Further, the leakage of cells from the beads was also observed. The major drawbacks of the immobilisation of microalgae reported in the literature included the occurrence of cell leakage from the polymeric matrix, the preservation of integrity and stability and biocompatibility [30,31,32]. For wastewater treatment purposes, natural matrices, such as alginate and carrageenan beads, are the most frequent choices. However, these matrices showed degradation and the strength of the beads were affected with the consequent effect on bioremediation efficiency. For the increase of the stability and integrity, the addition of synthetic polymers, such as PVA, have been suggested. Among the wide varieties of the available polymeric matrices, the choice of the polymer was driven by the reports on the increased stability of PVA in alginate beads, and by its biocompatibility. In the present study, a loss in the integrity of the beads was observed and the integrity of the beads was affected differently by the composition of the growth medium (nutrients and pharmaceuticals).

The results obtained from IBU, Figure 6, show the same behaviour. Immobilised cells removed IBU, from 50.4 to 44.3 μg mL^−1^ after 24 h of culture (*p* = 0.002 and F > F_crit_), and after 60 h of culture, IBU concentration apparently increased from 44.3 to 45.8 μg mL^−1^. The free cells group showed a slight removal of IBU, from 50.4 to 48.4 μg mL^−1^, after 60 h of culture.

The results from OLA display a different scenario. In particular, with the control group, f/2 medium, there was a drastic reduction from 49.0 to 33.1 μg mL^−1^ for the concentration of OLA after 12 h. All the groups were aerated, and it has previously been reported that this could affect the removal of some pollutants in the growth medium [33]. The same trend occurred in all the groups, a drastic reduction in the first 12 h, with a more pronounced reduction in the group of immobilised cells, from 49.0 to 29.9 μg mL^−1^, and in the free cells group, from 49.0 to 19.8 μg mL^−1^. Interestingly, after 12 h of culture, in the f/2 medium, beads and immobilised groups, the concentration of OLA remained unchanged. In the group of free cells, the concentration of OLA reached 12.2 μg mL^−1^ after 60 h of culture, suggesting more affinity with the molecule of OLA than with PAR and IBU, presumably reflecting the electrostatic interactions and other forces between them and the components of the membrane.

The method of cell immobilization is commonly used for enzymes, but not very common for microalgae. Some of the most common used matrices include natural polymers, such as alginate, agarose, chitosan and carrageenan. However, these natural polymers presents some disadvantages, such as low mechanical strength [34,35] (Jia and Yuan, 2016; Willaert and Baron, 1996). Synthetic polymers are considered to be more suitable for wastewater treatment and are considered to have a better mechanical performance compared to natural polymers (Cohen, 2001). However, the present study revealed the loss of stability of the PVA polymeric beads with the consequent release of the pollutant and cells from the matrix.

## 4. Conclusions

The microalga *Nannochloropsis* sp. was found to remain alive in the presence of the four pharmaceuticals, and to remove PAR, IBU and OLA from water, although with different efficiencies.

The results showed that free microalgae cells, when compared to the immobilized ones, remain alive for a longer period of time. However, the removal of paracetamol and ibuprofen by *Nannochloropsis* sp., after 24 h of culture, was significantly higher in immobilised cells. It has been found that free cells show a better performance on the removal of olanzapine, suggesting more affinity with this molecule than to paracetamol and ibuprofen. Fluorescence microscope observations showed the internalisation of olanzapine in living cells. 

The immobilisation studies indicated that PVA beads have limited the growth of the cells. However, compared with the free cells, the immobilised ones showed a higher resilience in the presence of the pharmaceuticals. In what pertains to the integrity of the beads, the experiments revealed the dissolution of the PVA beads. This could cause other environmental issues, such as the release of PVA into the environment, together with the removed pollutants. Therefore, more research is required to improve the stability and integrity of the beads.

From the results obtained in this study, it became apparent that the microalga *Nannochloropsis* sp. could be considered to be a promising species in the removal of pharmaceuticals from effluents. In addition, *Nannochloropsis* sp. is a promising biobased feedstock, rich in lipids, carbohydrates and proteins. Microalgae are considered one of the most promising renewable resources for high value biobased products and its use can be extended in a number of ways.
